# Multi-omics profiling to identify early plasma biomarkers in pre-diagnostic pancreatic ductal adenocarcinoma: a nested case-control study

**DOI:** 10.1016/j.tranon.2024.102059

**Published:** 2024-07-16

**Authors:** Emmy Borgmästars, Benjamin Ulfenborg, Mattias Johansson, Pär Jonsson, Ola Billing, Oskar Franklin, Christina Lundin, Sara Jacobson, Maja Simm, Zelmina Lubovac-Pilav, Malin Sund

**Affiliations:** a Department of Diagnostics and Intervention/ Surgery, Umeå University, Umeå, Sweden; b School of Bioscience, Department of Biology and Bioinformatics, University of Skövde, Skövde, Sweden; c Genomic Epidemiology Branch, International Agency for Research on Cancer, Lyon, France; d Department of Chemistry, Umeå University, Umeå, Sweden; e Division of Surgical Oncology, Department of Surgery, University of Colorado School of Medicine, Aurora, CO, USA; f Department of Clinical Sciences/ Obstetrics and Gynecology, Umeå University, Umeå, Sweden; g Department of Surgery, University of Helsinki and Helsinki University Hospital, Helsinki, Finland

**Keywords:** Pancreatic neoplasms, miRNomics, Metabolomics, Proteomics, Risk

## Abstract

Pancreatic ductal adenocarcinoma (PDAC) is an aggressive disease with poor survival. Novel biomarkers are urgently needed to improve the outcome through early detection. Here, we aimed to discover novel biomarkers for early PDAC detection using multi-omics profiling in pre-diagnostic plasma samples biobanked after routine health examinations.

A nested case-control study within the Northern Sweden Health and Disease Study was designed. Pre-diagnostic plasma samples from 37 future PDAC patients collected within 2.3 years before diagnosis and 37 matched healthy controls were included. We analyzed metabolites using liquid chromatography mass spectrometry and gas chromatography mass spectrometry, microRNAs by HTG edgeseq, proteins by multiplex proximity extension assays, as well as three clinical biomarkers using milliplex technology. Supervised and unsupervised multi-omics integration were performed as well as univariate analyses for the different omics types and clinical biomarkers. Multiple hypothesis testing was corrected using Benjamini-Hochberg's method and a false discovery rate (FDR) below 0.1 was considered statistically significant.

Carbohydrate antigen (CA) 19-9 was associated with PDAC risk (OR [95 % CI] = 3.09 [1.31–7.29], FDR = 0.03) and increased closer to PDAC diagnosis. Supervised multi-omics models resulted in poor discrimination between future PDAC cases and healthy controls with obtained accuracies between 0.429–0.500. No single metabolite, microRNA, or protein was differentially altered (FDR < 0.1) between future PDAC cases and healthy controls.

CA 19-9 levels increase up to two years prior to PDAC diagnosis but extensive multi-omics analysis including metabolomics, microRNAomics and proteomics in this cohort did not identify novel early biomarkers for PDAC.

## Highlights

• CA 19-9 levels were associated with PDAC risk and was elevated closer to PDAC diagnosis.
• No single metabolite, microRNA, or protein was differentially altered between future PDAC cases and healthy controls.
• Supervised DIABLO and blockForest models resulted in poor discrimination between future PDAC cases and healthy controls .
• MOFA analysis indicated that most of the plasma variation correlated with potential confounders, such as sex and age.
• No unsupervised multi-omics factors correlated with tumor-specific characteristics.

## Introduction

Pancreatic ductal adenocarcinoma (PDAC) is the most common form of pancreatic cancer with a dismal 5-year overall survival of only 13 % in the United States [1]. Detecting PDAC at earlier disease stages is crucial to improve the overall survival, as most patients are currently diagnosed at a non-curative stage. Carbohydrate antigen 19-9 (CA 19-9) is the most validated clinically used biomarker for PDAC, but its use for early diagnosis is hampered by suboptimal sensitivity and specificity [2]. We and others have previously utilized prospectively collected pre-diagnostic plasma samples to explore early alterations connected to PDAC, such as CA 19-9 [[3], [4], [5]], microRNAs [6,7], metabolomics [[8], [9], [10], [11]], tissue polypeptide specific antigens (TPS) [12], and fasting glucose levels [13,14]. A four-metabolic signature in combination with CA 19-9 with an external area under curve (AUC) of 0.90 has previously been developed after PDAC onset and is currently being evaluated in a pre-diagnostic PDAC cohort [15]. Eight miRNAs, selected based on published research, were previously studied in the European prospective investigation into cancer and nutrition (EPIC) PDAC cohort. An AUC of 0.73 was achieved for the eight miRNA-score within five years before PDAC diagnosis [6]. We previously identified a 15-miRNA signature at PDAC diagnosis, however the panel had poor accuracy in pre-diagnostic plasma samples [7].

The aforementioned studies on PDAC risk focused on single omics types, but combining multiple omics types (multi-omics) could potentially increase prediction accuracy. It could also provide insights into disease etiology since multi-omics allows for a more holistic approach where interactions between different omics types can be taken into account. Multi-omics biomarker signatures for cancer screening have previously been developed, such as the CancerSEEK test for detection of eight cancer types, including PDAC [16]. CancerSEEK combines levels of circulating tumor DNA with eight proteins, achieving a sensitivity of approximately 70 % to detect pancreatic cancer. Furthermore, its use in early cancer detection is limited since the sensitivity for all cancer types combined decreased from 75 to 78 % at cancer stages II & III to 40 % in stage I.

The aim of this study was to identify novel biomarkers for early PDAC detection by using a multi-omics approach. We analyzed three omics modalities in plasma samples from a pre-diagnostic PDAC cohort; metabolomics, miRNomics, and proteomics.

## Materials and methods

### Ethical statement

This study was conducted in accordance with the Helsinki Declaration of 1975 and ethical approval was obtained from the ethical committee at Umeå University (diary number 09-175M [approved 2009-11-03], amendments 2010-191-32 [approved 2010-05-20], 2011-320-32M [approved 2011-10-11], 2012-191-32M [approved 2011-10-11], 2013-5-32M [approved 2013-01-23], 2015-169-32M [approved 2015-05-20]). Written informed consent was given by the participants before inclusion into the Northern Sweden Health and Disease Study (NSHDS, Umeå, Sweden) biobank or the upper gastrointestinal (UGI) biobank at the Surgery unit (Northern Sweden University hospital, Umeå, Sweden).

### Study design

A nested case-control study was designed using pre-diagnostic plasma samples from PDAC patients. The plasma samples were obtained from the NSHDS. Samples had been collected in EDTA tubes, frozen within one hour after blood collection and stored at −80 °C.

### Pre-diagnostic cohort

We included individuals from the NSHDS who later developed PDAC without previous history of malignancy. The analysis was restricted to samples collected within 2.3 years prior to diagnosis (Supplementary Fig. 1). One healthy control was matched to each future PDAC plasma sample by sex, age (±5.3 years), and sample date (±5.0 years).

### Diagnostic cohort

A smaller, diagnostic cohort was constructed to enable further investigations of findings at diagnosis. Six pancreatic cancer cases, six breast cancer cases and six matched controls were included from the UGI biobank. The subjects were matched by sex, age (±4.1 years), and sample date (±2.6 years). Plasma samples have been stored in EDTA tubes at −80 °C.

### Clinical variables and pre-diagnostic symptoms

Information on sex, age, body mass index (BMI), fasting blood glucose, smoking, Swedish oral moist snuff (snus), and sample date were derived from the NSHDS study. Clinical variables and pre-diagnostic PDAC symptoms reported up to six years prior to diagnosis were derived from medical records. A description of inclusion and exclusion criteria for pre-diagnostic symptoms can be found in Supplementary Table 1.

### Clinical biomarkers

The clinical biomarkers CA 19-9, carcinoembryonic antigen (CEA) and CA 15-3 were analyzed using Milliplex Multiplex assays for Luminex kit Human circulating biomarker panel 1 (Merck) in 6 µL plasma. Each sample was analyzed in duplicates. A coefficient of variation (CV) cutoff <15 % was used. Values from an extrapolated standard curve were used for samples outside the quantification range. Larger CV was allowed for samples below limit of detection (LOD). Values below LOD was replaced with the lowest measurement divided by two. One pancreatic cancer sample collected at diagnosis seemed to be contaminated by fat and subsequently removed from the milliplex run due to a high CV.

### Protein profiling

A total of 644 proteins were analyzed on seven target panels using multiplex proximity extension assay (PEA) by Olink® (Uppsala, Sweden). The following target panels were analyzed in 7 µL plasma: Metabolism, Inflammation, Cardiovascular III, Oncology II, Oncology III, Cardiometabolic, and Immune Response. Protein data was delivered on log_2_ scale as protein expression values (NPX).

### Metabolomics

Swedish Metabolomics Centre (Umeå, Sweden) performed untargeted metabolomics including liquid chromatography mass spectrometry (LC–MS) and gas chromatography-MS (GC–MS) in 100 µL plasma. The samples included in this study were analyzed using metabolomics in a previous study [17]. The exact overlap of LC-MS- and GC-MS-detected metabolic features is unknown since some features are unidentified. However, we refer to these metabolic features as ‘metabolites’ in the present study.

### MicroRNA profiling

HTG Edgeseq utilizes predesigned probes that bind to the miRNA of interest and sequences the probes. HTG Edgeseq analysis of miRNA whole transcriptome (2083 miRNAs) in 15 uL plasma was conducted at TATAA Biocenter AB (Göteborg, Sweden). The HTG Edgeseq analysis can measure miRNAs freely in plasma and in extracellular vesicles. Five negative controls, one positive control, and 13 housekeeping genes were run in parallel.

### Hemolysis assessment

Hemolysis can affect levels of microRNAs and metabolites, and was assessed using Nanodrop as described previously [18,19]. Plasma samples, as well as their matched case or control, with a hemolysis value >25 mg/dL were excluded in the microRNA and metabolomics data analyses, as well as in multi-omics integration analyses.

### Data pre-processing

Clinical biomarkers and metabolite concentrations raised by 1 were log2-transformed. Metabolite, protein, and biomarker variables were scaled to unit variance in univariate analysis prior to conditional logistic regression and multi-omics integration.

We applied the following data pre-processing steps for the PEA proteins:

1) For proteins present in more than one panel, proteins from the panel with most successful runs and highest number of samples above LOD was kept (*n* = 15 proteins).
2) Boxplots of proteins with >70 % missing values split by case and control status (*n* = 41 proteins) were reviewed and the proteins that appeared to differ between cases and controls (*n* = 6) were kept for statistical analyses.
3) Measurements below LOD were replaced with $LOD/\sqrt{2}$.
4) Assays that did not pass quality control were replaced with NA values.

Since the count value of the five negative controls (included in the miRNA HTG edgeseq panel) varied between the samples, we subtracted the maximum value of the five negative control probes from all miRNAs sample-wise. A value of zero was imputed for miRNAs with a negative count value after adjustment for the negative background. MicroRNAs with a row-wise average logCPM < 2.16, which corresponded to a row sum of 10 counts, were excluded in downstream analyses. MicroRNA counts were transformed using *rlog* function in DESeq2 prior to multi-omics integration analyses [20].

### Statistical analyses

Statistical analyses were performed in R Project for Statistical Computing (RRID:SCR_001905) version 4.3.1 [21]. We adjusted for multiple testing using the Benjamini-Hochberg's procedure separately for each omics modality [22]. Since this study is explorative, an FDR < 0.1 was considered statistically significant.

Univariate analyses of metabolites, olink proteins, and biomarkers were performed using conditional logistic regression. We performed univariate paired analyses of microRNAs using the R packages DESeq2 (RRID:SCR_015687) version 1.32.0 and edgeR version 3.34.1 (RRID:SCR_012802)) [20,23]. Proteins and metabolites were also analyzed using LIMMA (RRID:SCR_010943) R package version 3.56.2, which uses information inference between variables to increase the power of small sample sizes [24]. Pearson's correlation was used for correlating variables to smoking, snus use, or sex.

A supervised DIABLO model was generated consisting of two latent factors with ten variables from each omics block using mixOmics R package (RRID:SCR_016889) [25,26]. A multi-omics random forest model was built using blockForest R package version 0.2.6 [27]. Prior to blockForest analysis, missing values were imputed using k-Nearest Neighbors method (kNN) in the impute R package version 1.74.1 (RRID:SCR_024243) [28]. The classification performance of the DIABLO and blockForest models was assessed using 5-fold cross-validation keeping the matched case-control pairs together. Accuracy was obtained using caret R package version 6.0.94 (RRID:SCR_021138) [29].

We performed unsupervised multi-omics integration analyses using MOFA2 R package version 1.2.2 (RRID:SCR_022992) for all samples or pre-diagnostic PDAC samples only [30]. The latent MOFA factors were correlated to clinical or technical parameters using Spearman's correlation implemented in psych R package version 2.3.6 (RRID:SCR_021744) [31]. We obtained ENSG-IDs for each protein using the Uniprot Retrieve/ID mapping tool (https://www.uniprot.org/id-mapping) [32,33]. Gene set enrichment analysis (GSEA) for proteins was performed using clusterProfiler (RRID:SCR_016884) version 4.8.3.

Figures were generated using ggplot2 (RRID:SCR_014601) version 3.3.6 [34], viridis version 0.6.4 (RRID:SCR_016696) [35], ggpubr (RRID:SCR_021139) version 0.6.0 [36], Corrplot (RRID:SCR_023081) R package version 0.92 [37], and graphic design software Affinity designer (RRID:SCR_016952) version 1.10.5 (Serif Europe Ltd). The R packages dplyr (RRID:SCR_016708) version 1.1.3 and tidyr (RRID:SCR_017102) version 1.3.0 was used for data management [38,39].

For protein, metabolite, and microRNA candidates with nominal *P*-value <0.05, a systematic literature search was performed in Pubmed (https://pubmed.ncbi.nlm.nih.gov/) to identify previous research supporting these candidates as potential biomarkers in PDAC. The search string used was ‘X AND (``Pancreatic adenocarcinoma'' OR “Pancreatic cancer” OR “pancreas cancer” OR “pancreatic ductal adenocarcinoma”) AND (Plasma OR serum)’, where X is a protein, microRNA, or metabolite with nominal *P*-value <0.05.

## Results

### Patient characteristics & univariate analyses

Pre-diagnostic plasma samples from 37 PDAC patients collected up to 2.3 years prior to diagnosis and 37 matched healthy controls were included from the NSHDS study (Supplementary Fig. 1). The mean age of PDAC patients were 59.4 years with a mean time of 1.3 years between sampling and time of diagnosis (Table 1). Most PDAC patients had metastasized disease (stage IV) at diagnosis. Testosterone levels were significantly correlated with sex and the nicotine-related metabolites cotinine and hydroxycotinine were in very good agreement with questionnaire information on tobacco use (Supplementary Table 2). Previously identified associations between cornulin (CRNN) and snus use, as well as persephin (PSPN) and sex were verified in our cohort [40,41].

**Table 1:** Clinical characteristics of the pre-diagnostic PDAC cohort.

Variable	Cases (*n* = 37)	Controls (*n* = 37)
Age at sampling (years, mean ± SD)	59.4 (± 6.3)	59.2 (± 6.2)
Follow-up time (years, mean ± SD)^a^	1.3 (± 0.7)	18.5 (± 5.2)
Sex, *n* (%)		
Men	10 (27 %)	10 (27 %)
Women	27 (73 %)	27 (73 %)
Sample date (years range)	1991–2014	1991–2010
Smoking status^b^, *n* (%)		
Smoker	9 (24 %)	8 (22 %)
Former smoker	12 (32 %)	7 (19 %)
Non-smoker/no answer in questionnaire	16 (43 %)	22 (59 %)
Fasting status^b^, *n* (%)		
>4 h	21 (57 %)	22 (59 %)
0–4 h	16 (43 %)	15 (41 %)
Mean BMI (± SD)^c^	26.5 (± 4.5)	25.2 (± 3.4)
Age at diagnosis (years, mean ± SD)	60.7 (± 6.3)	NA
CA 19-9 at diagnosis, *n* (%)		
<37 kE/L	1 (3 %)	NA
≥37 kE/L	6 (16 %)	NA
Information missing	30 (81 %)	NA
CEA at diagnosis, *n* (%)		
<2.5 μg/L	2 (5 %)	NA
≥2.5 μg/L	15 (41 %)	NA
Information missing	20 (54 %)	NA
Median survival (months)	5.7	NA
TNM stage at diagnosis, *n* (%)		
I	4 (11 %)	NA
II	6 (16 %)	NA
III	3 (8 %)	NA
IV	24 (65 %)	NA
Surgical treatment, *n* (%)		
None	23 (62 %)	NA
Curative intent	5 (14 %)	NA
Palliative intent	9 (24 %)	NA

a Time from blood collection to pancreatic cancer diagnosis for cases, time to end of follow-up for controls.

b Non-significant difference by Fisher's exact test.

c Non-significant difference by Student's *t*-test. SD = standard deviation, NA = not applicable, TNM = tumor-node-metastasis

**Fig. 1:**
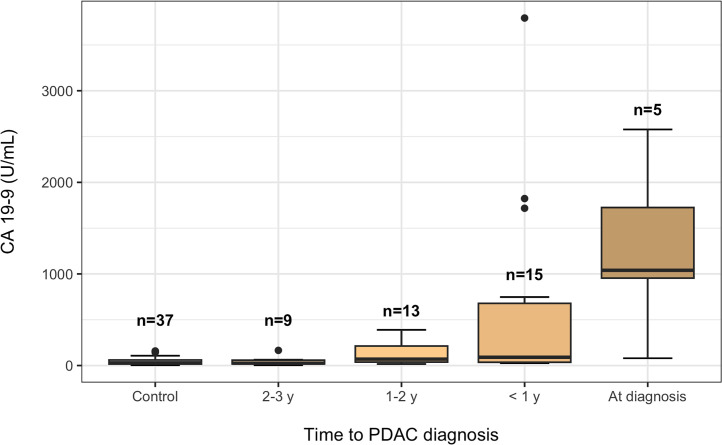
Boxplot of CA 19-9 plasma levels in relation to time to PDAC diagnosis. All samples belong to the pre-diagnostic cohort except for the category “At diagnosis”, which constitutes the smaller diagnostic PDAC cohort. The median of each group is represented by a solid black line. The lower and upper hinges of the box represent the 25th and the 75th percentile, respectively. Whiskers represent the minimum and maximum values within 1.5 times the lower and upper hinge, respectively. Outliers are shown as individual points. PDAC = pancreatic ductal adenocarcinoma.

The clinical biomarkers CA 19-9 (gold standard for PDAC), CEA, and CA 15-3 were analyzed in parallel to the omics modalities. CA 19-9 was associated with PDAC risk (OR for 1 standard deviation increase [95 % CI] = 3.03 [1.30–7.07], FDR = 0.031, Table 2). A trend with higher CA 19-9 levels closer to diagnosis was observed (Fig. 1). CA 19-9 levels increases were noted at 1–2 years prior to PDAC diagnosis and at three years prior to death (Supplementary Fig. 2). In addition to the PDAC risk, CA 19-9 levels were also correlated with snus status and CEA levels (Supplementary Fig. 3A).

**Table 2:** Odds ratios of biomarkers. Conditional logistic regression models were adjusted for body mass index, fasting status, and smoking status (37 cases and 37 controls).

	Crude model			Adjusted model		
Biomarker	OR (95 % CI)	*P*-value	FDR	OR (95 % CI)	*P*-value	FDR
CA19-9	3.03 (1.30–7.07)	0.010	0.031	7.32 (1.64–32.6)	0.014	0.043
CEA	1.19 (0.71–2.01)	0.512	0.769	1.14 (0.60–2.18)	0.683	0.683
CA15-3	1.05 (0.66–1.66)	0.846	0.846	0.81 (0.45–1.47)	0.501	0.683

OR = odds ratio per one standard deviation increase, FDR = false discovery rate

In univariable analyses of multi-omics, no microRNA, metabolite or protein was differentially expressed between future PDAC cases and healthy controls. However, our top candidates with a nominal *P*-value <0.05 that have previously been studied in PDAC were upregulated CPA1, ANG, miR-654-3p, and miR-6880-5p as well as downregulated cholesterol (D7) (Supplementary Table 3).

### Multi-omics integration

#### Discriminative ability between future pancreatic cancer cases and healthy controls

**Fig. 2:**
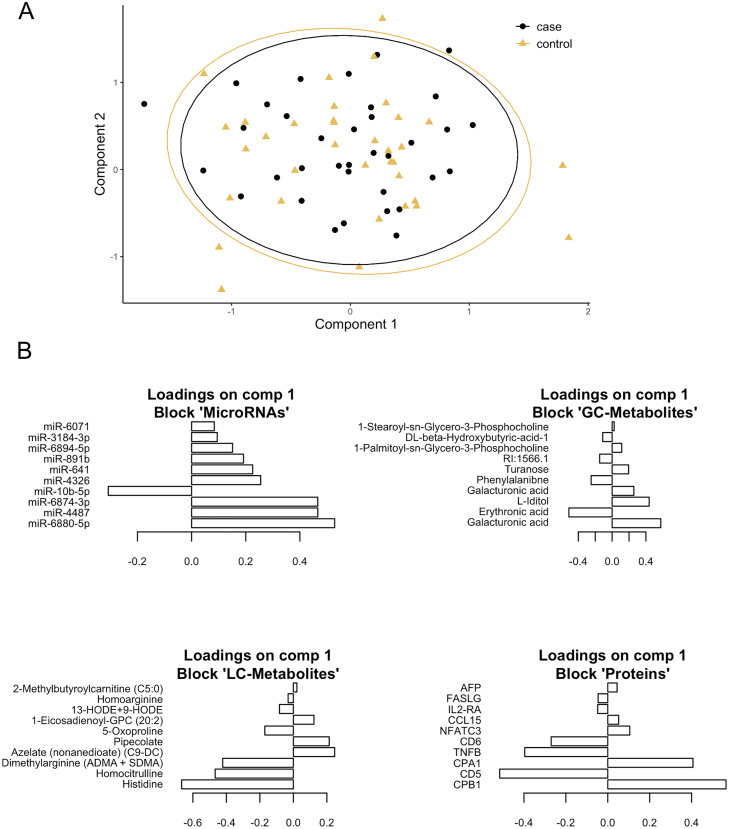
Supervised multi-omics DIABLO model separating between cases and controls. A) Visual separation between future PDAC cases and healthy controls based on DIABLO-generated components 1 and 2 obtained from 5-fold cross-validation. B) Top loadings for component 1. RI = retention index.

Three clinical biomarkers, 213 LC-metabolites, 197 GC-metabolites, 594 olink proteins, and 1987 HTG Edgeseq miRNAs were included in statistical models with the aim to identify a multi-omics biomarker signature able to distinguish between future PDAC cases and healthy controls. We constructed a supervised multi-omics model using DIABLO, including all microRNAs, metabolites, and proteins. The DIABLO model performed poorly with an accuracy of 0.500 (Fig. 2). The proteins block had the highest prediction accuracy (0.571) compared to the other omics blocks (Table 3). A multi-omics random forest model was generated using blockForest with an accuracy of 0.429.

**Table 3:** Accuracy of DIABLO model for the different omics blocks after 5-fold cross-validation.

Omics block	Accuracy
MicroRNAs	0.486
LC-Metabolites	0.371
GC-Metabolites	0.427
Proteins	0.571

LC = liquid chromatography, GC = gas chromatography

#### Exploring plasma multi-omics variance

**Fig. 3:**
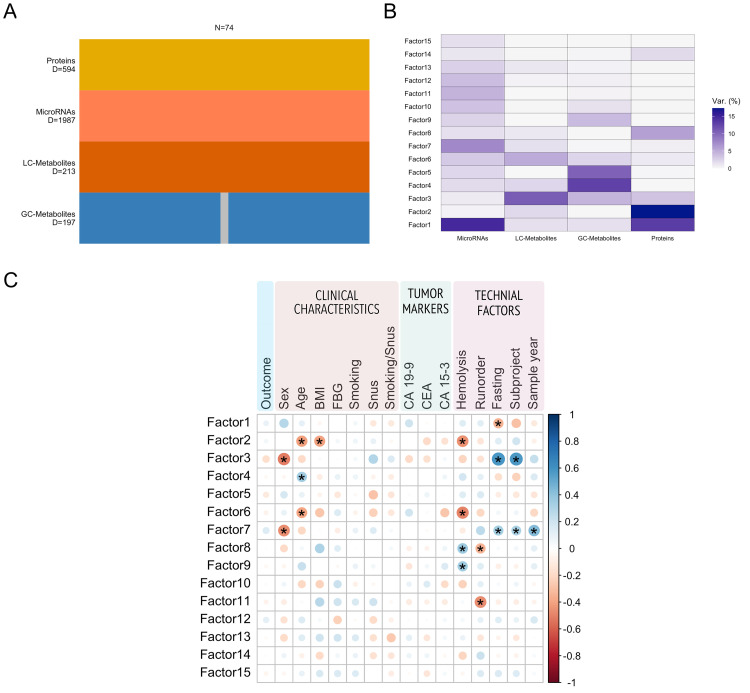
Unsupervised MOFA model. A) Number of samples and variables per omics block. Grey color indicates samples with missing values. B) Variance explained (%) for each factor split by omics block. C) Spearman's correlation between latent factors and clinical parameters. BMI = body mass index, FBG = fasting blood glucose, snus = Swedish oral moist snuff, CA 19-9 = carbohydrate antigen 19-9, CEA = carcinoembryonic antigen. * FDR < 0.1.

**Fig. 4:**
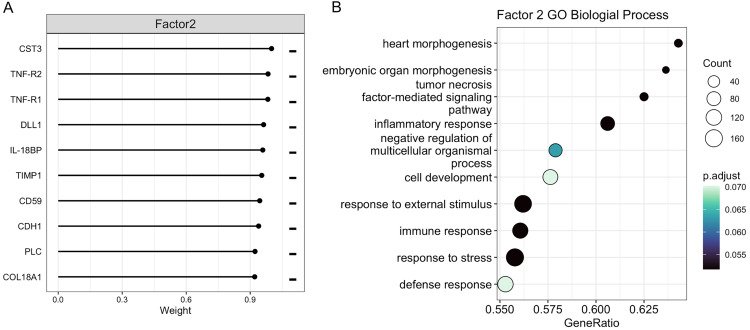
Gene set enrichment analysis of proteins in MOFA latent factor 2. A) Top 10 protein loadings in factor 2. The minus signs to the right indicate that all top ten proteins influence the latent factor negatively. B) Significantly enriched gene ontology (GO) biological process terms for latent factor 2 proteins (FDR < 0.1).

MOFA is an unsupervised multi-omics method similar to principal component analysis that can identify major drivers of variation while simultaneously providing the possibility to explore the variance specific to different omics types [30]. Fifteen latent factors were modelled based on microRNAs, metabolites, and proteins (Fig. 3). The number of variables in each omics view as well as the variance explained for each latent factor is shown (Fig. 3A and B). Latent factors 1–11 were correlated with different clinical or technical covariates but not with the outcome of interest (PDAC or healthy) (Fig. 3C). Our matching factors sex, age, and sample year were correlated with latent factors 2, 3, 4, 6, and 7. Fasting status, subproject, and sex are highly correlated since one of the subprojects in NSHDS only included women. In addition, fasting status differed between the subprojects, where all Mammography participants (*n* = 30) have a fasting time of 0–4 h, whereas the majority of Västerbotten Intervention Programme (VIP) participants (39/41) have a fasting time ≥ 8 h. Obesity and smoking are known PDAC risk factors but no latent factor was correlated with smoking status, and only factor 2 was correlated with BMI. Most of the variance explained in latent factor 2 can be attributed to proteins (17.3 %) and Cystatin-C (CST3) had the strongest influence on latent factor 2 (Fig. 4A). Gene set enrichment analysis of protein loadings in latent factor 2 resulted in gene ontology terms associated with morphogenesis, tumor necrosis factor-mediated signaling, different types of responses (Fig. 4B).

**Fig. 5:**
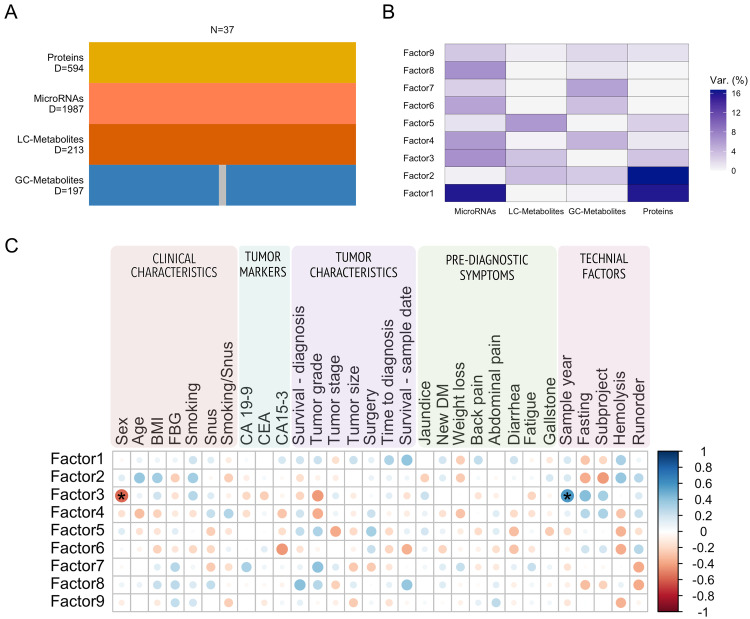
Unsupervised MOFA model for pre-diagnostic PDAC cases. A) Number of samples and dimensions per omics block. Grey color indicates samples with missing values. B) Variance explained (%) for each factor split by omics block. C) Spearman's correlation between latent factors and clinical, tumor, symptoms, or technical parameters. BMI = body mass index, FBG = fasting blood glucose, snus = Swedish oral moist snuff, CA 19-9 = carbohydrate antigen 19-9, CEA = carcinoembryonic antigen, Survival—diagnosis = time between diagnosis and death, Survival—sample date = time between sample date and death. * FDR < 0.1.

A separate MOFA model was generated containing only the pre-diagnostic PDAC cases in order to include tumor specific characteristics at PDAC diagnosis and reported symptoms up to six years prior to diagnosis (Fig. 5, Supplementary Table 4). The number of variables in each omics view as well as the variance explained for each latent factor is shown (Fig. 5A and B). Latent factor 3 was correlated with the covariates sex and sample year (FDR < 0.1, Fig. 5C). No latent factor correlated with tumor-specific characteristics, such as tumor stage or grade, nor the PDAC biomarker CA 19-9. Also, latent factors 1 and 2 explain a larger fraction of variance compared to latent factor 3, but do not correlate to any of the included covariates. The results suggest that the variance in these pre-diagnostic PDAC samples might be associated with unmeasured covariates.

## Discussion

Manifest PDAC is associated with broad plasma alterations in metabolites, miRNAs and proteins but less is known regarding early alterations and associations with PDAC risk [[42], [43], [44]]. Here, we performed a wide multi-omics screening by analyzing microRNAs, metabolites, and proteins in pre-diagnostic PDAC plasma samples to identify potential biomarkers for early PDAC detection. The gold standard PDAC biomarker CA 19-9 was elevated up to two years before a PDAC diagnosis. We identified no strong early PDAC signal based on circulating proteins, miRNAs, or metabolites up to 2.3 years before a PDAC diagnosis. Unsupervised MOFA factors showed stronger correlation with other clinical characteristics rather than with the outcome or tumor characteristics.

Several studies suggest a time window of several years between the initiating tumor or pre-cancerous lesions and PDAC [[45], [46], [47]]. One study based on mutation profiles of paired primary PDAC and metastases suggested a time window of 10 years from initiating tumor to first PDAC founder cell and another five years until metastasized cancer, implying early PDAC detection is possible [45]. In contrast, chromothripsis seems to be a frequent event in PDAC indicating a rapid development of PDAC [48,49]. A comparison of patient age at PDAC stages I versus IV suggested a fast progression of 1.3 years on average from early to metastasized disease [50]. We found that the gold standard PDAC biomarker CA 19-9 [51,52] was associated with an increased PDAC risk and starts to increase within two years prior to PDAC diagnosis (Fig. 1, Table 1) and within three years prior to death (Supplementary Fig. 2). Our findings are supported by previous research of elevated circulating CA 19-9 levels in pre-diagnostic PDAC samples [[3], [4], [5],^53^]. In a previous study using a cohort that partly overlaps with ours, CA 19-9 was observed to increase up to two years prior to PDAC diagnosis [3]. In a pre-diagnostic PDAC cohort of postmenopausal women, O'Brien et al found serum CA 19-9 to be differentially expressed between cases and controls up to two years prior to diagnosis [5]. Honda and coworkers reported an increase in plasma CA 19-9 levels in PDAC patients 18 months prior to diagnosis in plasma samples derived from the EPIC study [53]. Serum CA 19-9 levels were differentially altered between pre-diagnostic PDAC cases and controls up to two years prior to diagnosis in a cohort derived from the Prostate, Lung, Colorectal and Ovarian (PLCO) Cancer Screening Trial [4].

Neither univariate nor multi-omics statistical analyses pointed towards a clear, early circulating signal in our pre-diagnostic PDAC cohort. This could be due to small sample size and heterogeneous samples, or it may indicate that the cancer-related signal is not sufficiently strong up to 2.3 years prior to diagnosis. Cholesterol (D7, retention index 3146.6), CPA1, ANG, miR-654-3p, and miR-6880-5p, each showing a nominal *P*-value <0.05 have previously been studied in PDAC and could be interesting to study in external PDAC cohorts (Supplementary Table 3). The branched chain amino-acids (BCAA) isoleucine, leucine, and valine have previously been found associated with PDAC risk at 2–5 years [10] and ≥ten years prior to PDAC diagnosis [9]. We did not identify any of the BCAA as associated with PDAC risk in our study but found significant correlations to other parameters, such as smoking status, sex, age, and hemolysis degree (Supplementary Fig. 3B). Another metabolomics study in five pre-diagnostic PDAC cohorts did not identify differentially altered plasma metabolites after adjusting for multiple testing, which is in line with our results [8]. No plasma protein associations were found in pre-diagnostic pancreatic cancer in the UK Biobank Pharma Proteomics Project (UKB-PPP), which is similar to our results [54]. Furthermore, we were not able to replicate the findings from a case-subcohort study in a Chinese population with 15 differentially expressed proteins (from the Olink immuno-oncology panel) associated with PDAC risk within nine years before diagnosis [55]. Five proteins (CD4, HMOX1, GZMA, CRTAM, and ADGRG1) were not included in our selected Olink panels and two proteins (IL2 and IL4) were excluded from statistical analyses due to a high frequency of < LOD measurements (89–100 %). The remaining eight proteins (MCP-3, ANGPT2, IL18, IL6, LAMP3, CCL3, CD8A, and HGF) were not significantly altered between future PDAC cases and healthy controls in our study. One possible explanation for this difference could be the longer follow-up time of nine years in the referenced study compared to 2.3 years in our study. Also, the ethnicity could have an impact on this difference. For the subcohort, Kartsonaki et al randomly selected a subset of the baseline cohort that resulted in differences in age and sex between PDAC cases and controls, however the models were adjusted for potential confounders including sex and age. In our data, MCP-3, IL18, Il6, CCL3, and HGF correlated to BMI and/or hemolysis degree (Supplementary Fig. 3C). Among the previously identified differentially expressed miRNAs in a pre-diagnostic PDAC cohort [6], no significant correlations were found with clinical variables (Supplementary Fig. 3D). None of the latent factors generated by MOFA correlated to tumor-specific characteristics, such as tumor stage or grade, which supports a late-arising PDAC or little systemic effect from an existing tumor.

Clinical samples can exhibit large heterogeneity due to technical or biological factors, such as sample handling, storage time, disease, comorbidities, risk factors, and lifestyle factors. In the NSHDS study, plasma samples are frozen within one hour after sampling and stored at −80 °C, which mitigates technical variation associated with sample handling and storage, thereby ensuring high sample quality. By correlating the unsupervised MOFA factors with clinical variables, we observed that the latent MOFA factors showed stronger correlation to other clinical variables, such as smoking status, BMI, and age, rather than the outcome of interest. Some of the latent factors correlated to our matching covariates (sex, age, and sample year), which highlights the advantage of a matched case-control design to mitigate potential confounding effect.

The major strength in this study is the extensive information we have on participating individuals, particularly the PDAC patients, including their reported pre-diagnostic symptoms. Pre-diagnostic samples are extremely valuable, and the total plasma volume needed to analyze 2991 variables (microRNAs, metabolites, and proteins) was as low as 122 μL. The different omics modalities were performed on the same samples, allowing for integrated multi-omics analyses. One challenge in this study is the heterogeneity of our cohort due to differences in time to diagnosis as well as other clinical variables. In addition, the PDAC patients exhibited different tumor characteristics at the time of diagnosis.

In conclusion, plasma CA 19-9 levels increase up to two years prior to PDAC diagnosis. No multi-omics signal or single miRNA, metabolite, or protein deviation that precedes a PDAC diagnosis was identified up to 2.3 years prior to diagnosis.

## Disclaimer

Where authors are identified as personnel of the International Agency for Research on Cancer / World Health Organization, the authors alone are responsible for the views expressed in this article and they do not necessarily represent the decisions, policy or views of the International Agency for Research on Cancer / World Health Organization.

## CRediT authorship contribution statement

**Emmy Borgmästars:** Writing – original draft, Visualization, Investigation, Funding acquisition, Formal analysis, Conceptualization. **Benjamin Ulfenborg:** Writing – review & editing, Supervision, Formal analysis, Conceptualization. **Mattias Johansson:** Writing – review & editing, Conceptualization. **Pär Jonsson:** Writing – review & editing, Supervision, Formal analysis, Data curation, Conceptualization. **Ola Billing:** Writing – review & editing, Supervision, Conceptualization. **Oskar Franklin:** Writing – review & editing, Supervision, Project administration, Funding acquisition, Conceptualization. **Christina Lundin:** Investigation. **Sara Jacobson:** Investigation. **Maja Simm:** Investigation. **Zelmina Lubovac-Pilav:** Writing – review & editing, Supervision, Conceptualization. **Malin Sund:** Writing – review & editing, Supervision, Project administration, Funding acquisition, Conceptualization.

## Declaration of competing interest

Emmy Borgmästars received funding from The JC Kempe Memorial Foundation Scholarship Fund. Oskar Franklin received funding from The Royal Swedish Academy of Sciences (PE Lindahl Foundation, LM2021-0010 and LM2023-0012), The Swedish Society of Medicine (SLS-960379), Region Västerbotten in Umeå, Sweden (RV 967602) Cancerforskningsfonden i Norrland (LP 23-2337), Bengt Ihre foundation (SLS-960529 and SLS-986656) and Bengt Ihre Research Fellowship Grant. Malin Sund received funding from Umeå University, the Swedish Research Council [2016-02990, 2019-01690], the Swedish Cancer Society [CAN 2016/643, 19 0273], Västerbotten Region [RV-583411, RV-549731, RV-583411, RV-841551], Finska Läkaresällskapet, Medicinska Understödsföreningen Liv och Hälsa, and the Sjöberg foundation. The other authors declare no competing interests.

## Data Availability

Data is not freely available due to personal privacy reasons (according to General Data Protection Regulation). Data may be available upon reasonable request and provided that an ethical approval has been granted.

## Appendix Supplementary materials

Image, application 1
