# Supplementary material for: Multi-omics profiling to identify early plasma biomarkers in pre-diagnostic pancreatic ductal adenocarcinoma: a nested case-control study
Source: Transl Oncol. 2024 Jul 16;48:102059. doi: 10.1016/j.tranon.2024.102059 (PMC11301391; doi:10.1016/j.tranon.2024.102059)
Supplement: Supplementary file 1 [file mmc1.docx]

# Supplementary information

## Supplementary Methods

**Supplementary Table 1. Description of inclusion and exclusion criteria of reported pre-diagnostic PDAC symptoms.**

| Symptom | Inclusion | Exclusion |
| --- | --- | --- |
| Abdominal pain | Stinging, burning, dull pain sensation, abdominal discomfort, or general abdominal pain | Diffuse abdominal problems |
| Back pain | Lumbago, general back pain, thoracic back pain, or pain that radiates towards the back | Strict cervical back pain |
| Fatigue | General fatigue | - |
| Diarrhea | Stated diarrhea | - |
| Weight loss | Described weight loss | - |
| Jaundice | Stated jaundice or yellow sclerae or skin | - |
| Newly diagnosed diabetes mellitus | Diabetes mellitus diagnosis ≤ three years before PDAC diagnosis | Increased blood glucose only, previous diabetes mellitus diagnosis |
| Gallstone | Gallstone diagnosis | - |

## Supplementary Results


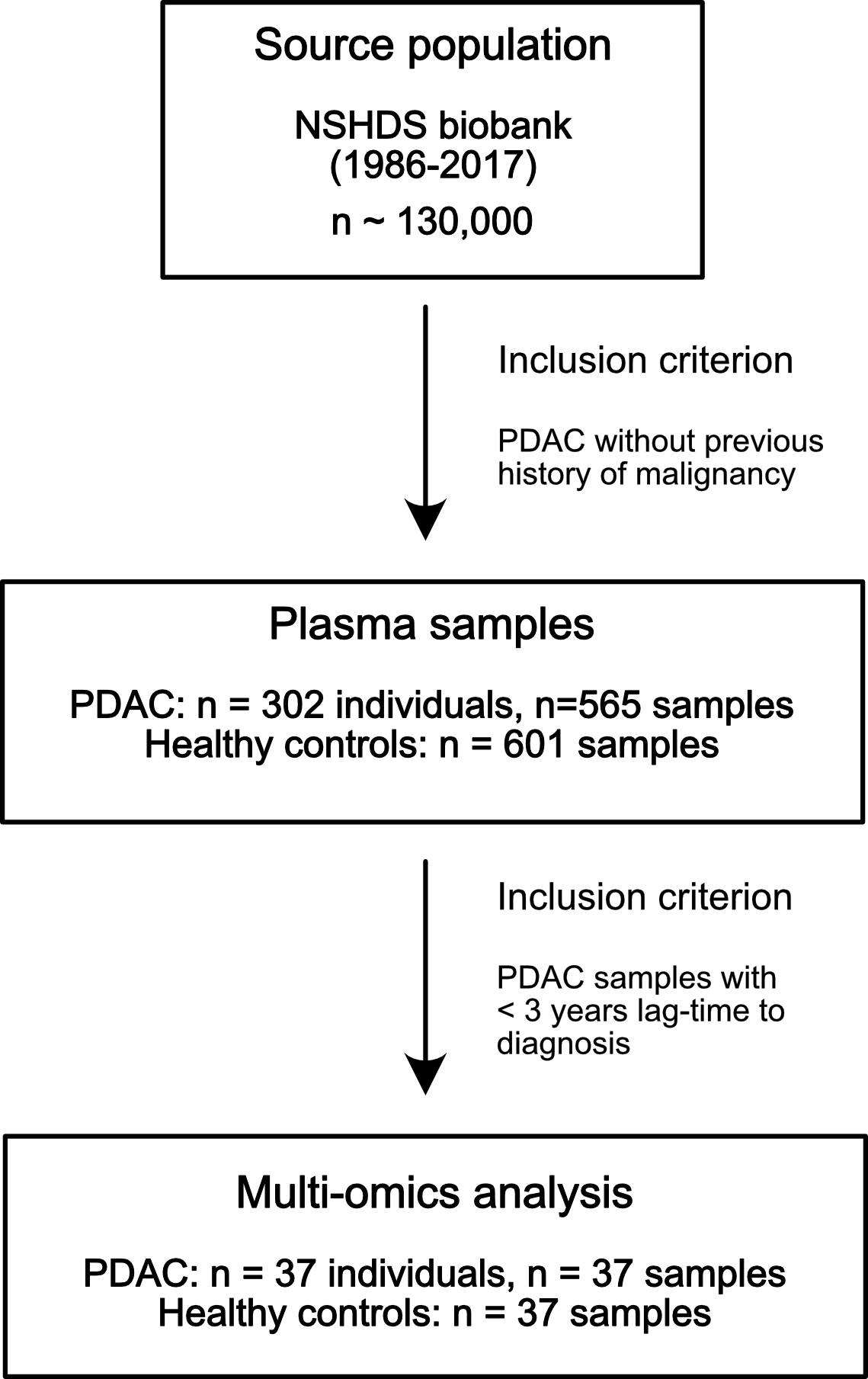


**Supplementary Figure 1.** **Flowchart of included individuals in the pre-diagnostic PDAC cohort.**


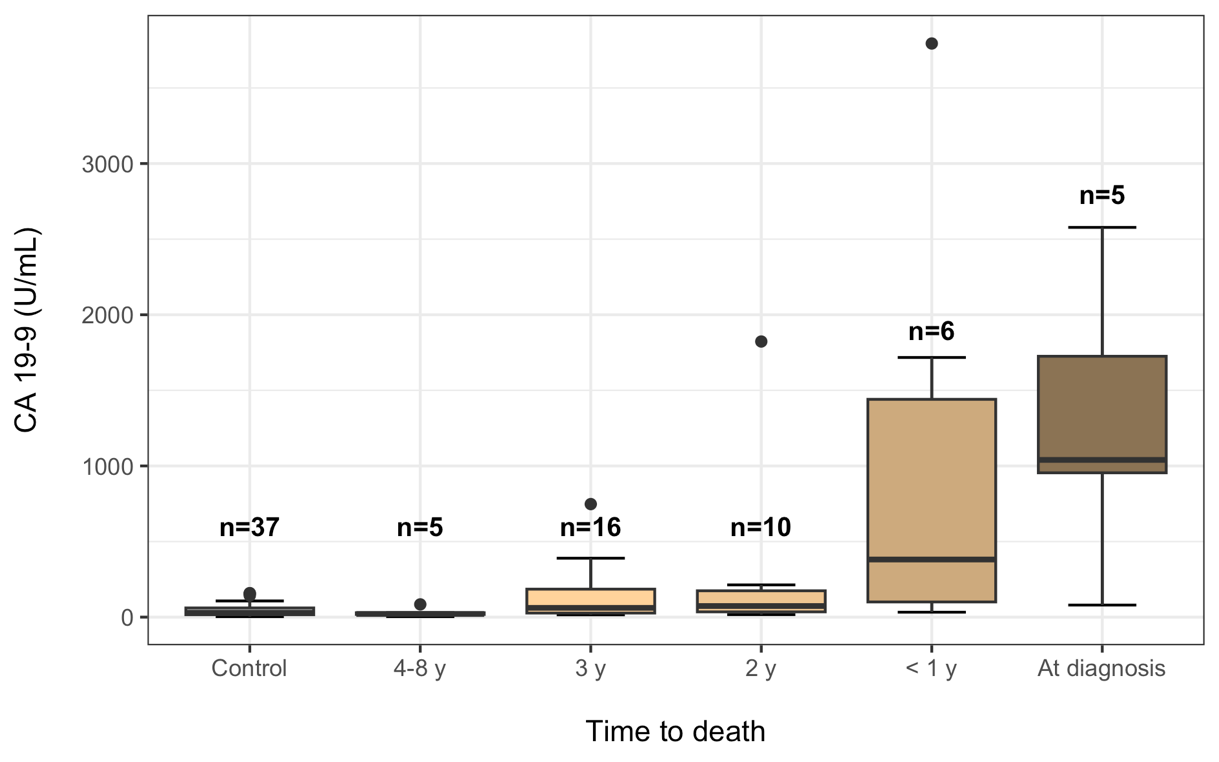


**Supplementary Figure 2.** **Boxplot of CA 19-9 levels in relation to time between sample date and death.** All samples belong to the pre-diagnostic cohort except for the category “At diagnosis”, which constitutes the smaller diagnostic PDAC cohort. The median of each group is represented by a solid black line. The lower and upper hinges of the box represent the 25^th^ and the 75^th^ percentile, respectively. Whiskers represent the minimum and maximum values within 1.5 times the lower and upper hinge, respectively. Outliers are shown as individual points. PDAC = pancreatic ductal adenocarcinoma.

**Supplementary Table 2.** Associations between selected patient characteristics and multi-omics variables performed as data sanity checks or verification of previous findings.

| **Patient characteristic** | **Multi-omics variable** | **PCC** | **P-value** |
| --- | --- | --- | --- |
| Sex^1^ | Testosterone | 0.86 | < 0.001 |
| Sex^1^ | PSPN | 0.79 | < 0.001 |
| Snus or smoking status^2^ | Cotinine | 0.89 | < 0.001 |
| Snus or smoking status^2^ | Hydroxycotinine | 0.90 | < 0.001 |
| Snus status^2^ | CRNN | 0.38 | 0.002 |

^1^ Female = 0, male = 1

^2^ Non user = 0, user = 1

PCC = Pearson’s correlation coefficient, PSPN = persephin, snus = Swedish oral moist snuff, CRNN = cornulin

**Supplementary Table 3. Proteins and microRNAs with nominal P-value < 0.05 in relation to previous research of circulating levels in association with PDAC.** An OR of 1 = no effect, larger than 1 = upregulated in PDAC, below 1 = downregulated in PDAC, whereas logFC or log_2_FC of 0 indicates no effect, larger than 0 = upregulated in PDAC, and below 0 = downregulated in PDAC.

| **Variable** | **Statistical method** | **↑↓** | **Effect size** | **P-value** | **FDR** | **Previous research** |
| --- | --- | --- | --- | --- | --- | --- |
| (Most likely) Cholesterol (D7),  RI = 3146.6 | clogit | down | OR (95 % CI),  0.41 (0.17-0.98) | 0.046 | 0.970 | Lower PDAC risk with high total cholesterol ≥240 mg/dL in the recent three years before PDAC diagnosis (OR = 0.50, 95% CI 0.27–0.93) [1].  Lower total serum cholesterol associated with reduced PDAC risk [2]. |
|  | LIMMA^1^ | down | logFC, -0.21 | 0.033 | 0.974 |  |
| CPA1 | clogit | up | OR (95 % CI),  2.02 (1.13-3.60) | 0.017 | 0.957 | Serum CPA *activity* + CA 19-9 increased diagnostic utility in PDAC without atrophy compared to CA 19-9 alone [3] |
|  | LIMMA^1^ | up | logFC, 0.57 | 0.006 | 0.621 |  |
| ANG | clogit | up | OR (95 % CI),  2.20 (1.07-4.51) | 0.031 | 0.957 | Higher plasma or serum levels in PDAC [4, 5] |
|  | LIMMA^1^ | up | logFC, 0.20 | 0.021 | 0.965 |  |
| miR-654-3p | DESeq2^1^ | up | log_2_FC, 1.393 | 0.008 | 1.000 | Downregulated serum levels in PDAC at diagnosis (opposite to our results) [6] |
|  | edgeR^1^ | up | logFC, 1.290 | 0.004 | 1.000 |  |
| miR-6880-5p | DESeq2^1^ | up | log_2_FC, 0.924 | 0.009 | 1.000 | Downregulated serum levels in PDAC at diagnosis (opposite to our results) [7] |
|  | edgeR^1^ | up | logFC, 0.862 | 0.048 | 1.000 |  |

FDR = false discovery rate, RI = retention index, clogit = conditional logistic regression, OR = odds ratio per standard deviation increase, CI = confidence interval, logFC = log fold change, CPA1 = carboxypeptidase A1, ANG = angiogenin

^1^ R package

**Supplementary Table 4. Patients with pre-diagnostic symptoms and a blood sample collected at the same time or after reported symptom.** One patient had no information.

| **Symptom** | **Patients with reported symptom (%)** |
| --- | --- |
| Abdominal pain | 36 |
| Back pain | 22 |
| Fatigue | 17 |
| Diarrhea | 11 |
| Weight loss | 6 |
| Jaundice | 3 |
| Newly diagnosed diabetes mellitus | 3 |
| Gallstone | 3 |


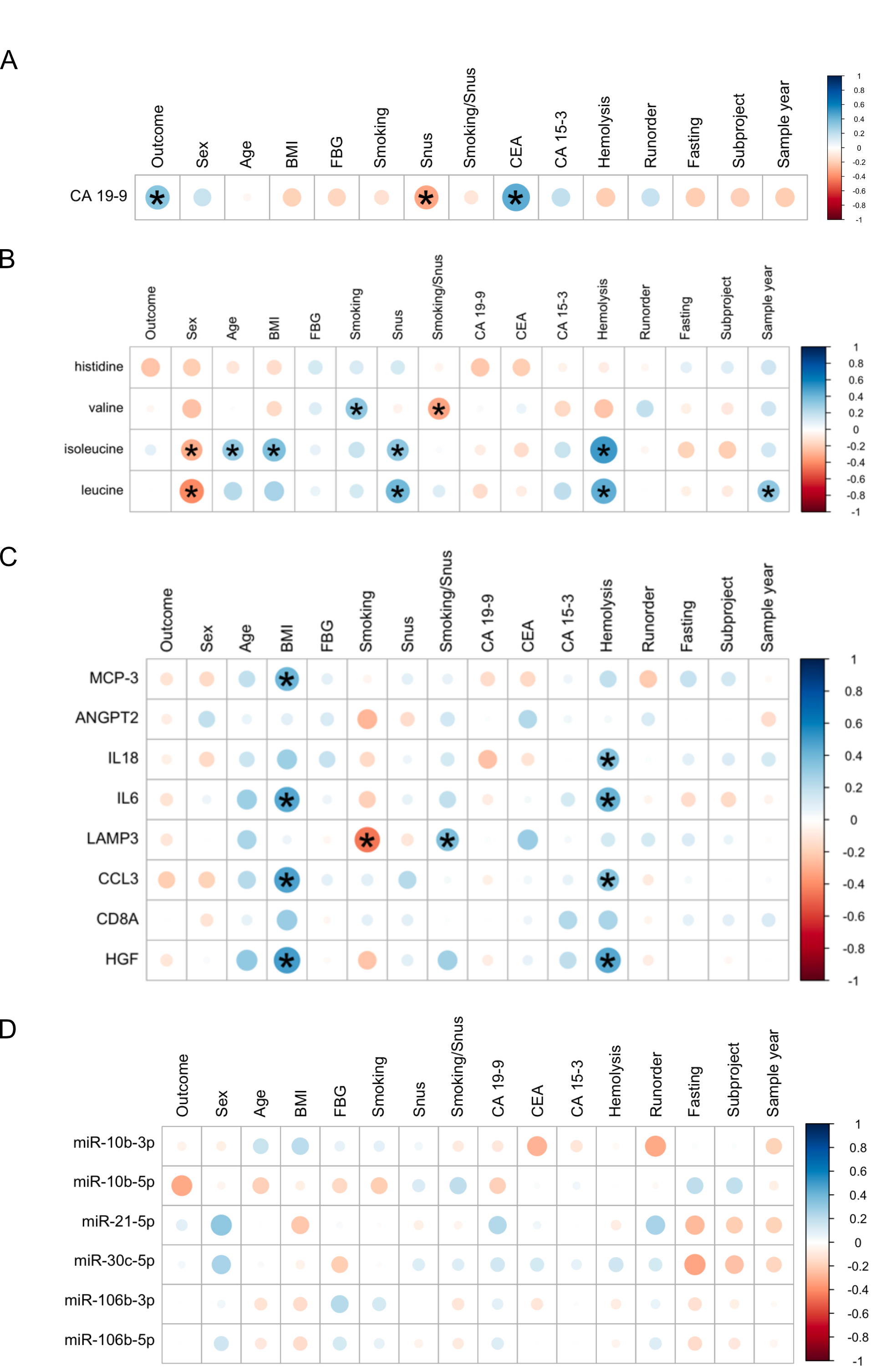


**Supplementary Figure 3. Spearman’s correlation coefficient between selected variables and clinical or technical parameters**. A) Correlation between CA 19-9 and clinical or technical parameters. B) Correlation between selected metabolites and clinical or technical parameters. C) Correlation between selected proteins and clinical or technical parameters. D) Correlation between selected microRNAs and clinical or technical parameters. BMI = body mass index, FBG = fasting blood glucose, CA 19-9 = carbohydrate antigen 19-9, CEA = carcinoembryonic antigen * FDR < 0.1

## References

1. Wang, Q.L., et al., *Temporal Association of Total Serum Cholesterol and Pancreatic Cancer Incidence.* Nutrients, 2022. **14**(22).

2. Chen, W.C., et al., *Total Serum Cholesterol and Pancreatic Cancer: A Nested Case-Control Study.* Cancer Epidemiol Biomarkers Prev, 2019. **28**(2): p. 363-369.

3. Tanaka, H., et al., *Serum Carboxypeptidase Activity and Genotype-Stratified CA19-9 to Detect Early-Stage Pancreatic Cancer.* Clin Gastroenterol Hepatol, 2022. **20**(10): p. 2267-2275 e2.

4. Shimoyama, S., et al., *Increased angiogenin expression in pancreatic cancer is related to cancer aggressiveness.* Cancer Res, 1996. **56**(12): p. 2703-6.

5. Wang, Y.N., et al., *Angiogenin/Ribonuclease 5 Is an EGFR Ligand and a Serum Biomarker for Erlotinib Sensitivity in Pancreatic Cancer.* Cancer Cell, 2018. **33**(4): p. 752-769 e8.

6. Du, W., et al., *LINC01232 Sponges Multiple miRNAs and Its Clinical Significance in Pancreatic Adenocarcinoma Diagnosis and Prognosis.* Technol Cancer Res Treat, 2021. **20**: p. 1533033820988525.

7. Kojima, M., et al., *MicroRNA markers for the diagnosis of pancreatic and biliary-tract cancers.* PLoS One, 2015. **10**(2): p. e0118220.
